# Unmet supportive care needs in families of children with chronic health conditions: an Australian cross-sectional study

**DOI:** 10.1007/s12519-023-00730-w

**Published:** 2023-05-29

**Authors:** Sangeetha Thomas, Linda K. Byrne, Nicholas P. Ryan, Christel Hendrieckx, Victoria White

**Affiliations:** 1 School of Psychology, Faculty of Health, Deakin University, Melbourne, VIC 3125 Australia; 2 Department of Paediatrics, University of Melbourne, Melbourne, VIC Australia; 3 Australian Centre for Behavioural Research in Diabetes, Melbourne, VIC Australia; 4 Institute of Health Transformation, Deakin University, Melbourne, VIC Australia; 5 Cancer Council Victoria, Melbourne, VIC Australia

**Keywords:** Children, Chronic illness, Need assessment, Unmet supportive care needs

## Abstract

**Background:**

The aim of this study was to identify similarities and differences in the unmet supportive care needs (USCN) of families of children with major chronic health conditions (CHCs) using a universal need assessment tool.

**Methods:**

A cross-sectional online survey involving parents of children with congenital heart disease (CHD), type 1 diabetes mellitus (T1D), cancer, and asthma diagnosed within the last 5 years recruited via social media and support organizations. Thirty-four items assessing the USCN across six domains (care needs, physical and social needs, informational needs, support needs, financial needs, child-related emotional needs) were responded to on a 4-point Likert scale [no need (1) to high need (4)]. Descriptive statistics identified the level of need, and linear regressions identified factors associated with higher need domain scores. Due to small numbers, the asthma group was excluded from comparisons across CHCs.

**Results:**

One hundred and ninety-four parents completed the survey (CHD: *n* = 97, T1D: *n* = 50, cancer: *n* = 39, and asthma: *n* = 8). Parents of children with cancer were most likely to report at least one USCN (92%), followed by parents of children with T1D (62%). The five most commonly reported USCN across CHCs were drawn from four domains: child-related emotional, support, care, and financial. Three need items were included in the top five needs for all conditions. A higher USCN was associated with a greater frequency of hospital visits and the absence of parental support.

**Conclusions:**

Using a universal need assessment tool, this is one of the first studies to characterize USCN in families of children diagnosed with common CHCs. While proportions endorsing different needs varied across conditions, the most endorsed needs were similar across the illness groups. This suggests that support programs or services could be shared across different CHCs.

**Video Abstract**

**Supplementary Information:**

The online version contains supplementary material available at 10.1007/s12519-023-00730-w.

## Introduction

Chronic health conditions (CHCs) are those that last longer than a year, require regular medical attention, and cause limitations in daily life [1, 2]. In Australia, the prevalence of chronic disease is increasing, with recent data (2020–2021) indicating that approximately 44% of children have at least one CHC (inclusive of mental health conditions) [3, 4]. A CHC in childhood can have a lasting negative impact on educational, social, and emotional outcomes [5]. Medical care for children with CHC may include complex and/or ongoing treatment, with families requiring diligence, energy, and skills to manage their child’s health (e.g., ensuring medications taken as prescribed) [6]. Additionally, parents need to provide physical, emotional, and practical support to their child while maintaining work, family, and social roles [7–9].

Although the medical care of children with different chronic conditions varies, there is similarity in patterns of parental adjustment and experiences [9–16]. For instance, a systematic review identified common experiences of parents of children with different long-term conditions relating to a lack of information and support, managing emotional and physical burdens, mastering technical aspects of the child’s care, coordinating services and seeking social support systems [14]. A systematic review exploring father’s experiences caring for a child with a chronic illness found increased anxiety and depression, lower self-esteem and a need for social and informational support regardless of the health condition [16].

Supportive care needs (SCN) refer to the services or support that people with a health condition or those caring for them may need [17, 18]. The SCN framework, originally developed in relation to cancer care, classifies needs into a number of different domains, including physical, emotional, social, psychological, informational, spiritual, health care, and practical care [17, 19]. A gap in the support parents need and the support they receive reflects unmet supportive care needs (USCN), highlighting areas where further services are required [20–24]. Determining the USCN of parents caring for children with a CHC can help to develop programs and services to enhance parents wellbeing and assist children in their adjustment to their health condition [8, 25–27]. The SCN framework and concept have been extended to consider the needs of carers and families of children with life-limiting and rare conditions, including pediatric cancers [19, 23, 25, 28–32].

Research has demonstrated the universality of need domains across different groups [28, 31, 33]. However, as the majority of research examining USCN in families of children with a CHC has focused on cancer, our understanding of similarity and differences in specific needs across childhood CHCs is limited [26, 33]. Furthermore, the lack of a consistent method to assess unmet needs across different conditions [33] makes it difficult to determine whether there exists a common pattern of needs across families of children with different CHCs. To start to address this gap, this study aimed to use a universal need assessment to (1) characterize the unmet SCN across different common childhood CHCs; (2) compare frequencies of moderate/high needs across conditions; and (3) determine associations between level of need and parental demographic characteristics, child-specific factors, and illness-related variables.

## Methods

### Sample and setting

This study comprised an online cross-sectional survey involving a convenience sample of Australian parents of children with common CHCs. The survey was open between November 2020 and October 2022. Inclusion criteria were as follows: (1) Australian parents of children diagnosed with one of the following CHCs: congenital heart disease (CHD), type 1 diabetes mellitus (T1D), cancer, or asthma; (2) children aged between 0 and 18 years; and (3) receiving treatment or managing their condition in the last 5 years. Exclusion criteria included children with a genetic, developmental, cognitive, or mental health condition.

### Measures

Parents completed an online survey consisting of items measuring the USCN, child-illness factors, and demographic characteristics of the child and family.

#### Unmet supportive care needs survey

Given that there currently exists no universal instrument to assess USCN in families of children with different CHCs [33], our approach was guided by Denham et al.’s (2020) framework for assessing USCN in carers of adults with mixed CHCs [34]. We developed a set of items drawing on USCN scales developed for cancer: eight items from the Family Inventory of Needs–Pediatric II [35] and thirteen items from the Cancer Survivors Unmet Needs scale [36]. Fourteen additional items derived from the CHC literature were included in the survey. Items were designed to cover five domains identified, with the addition of items assessing financial needs [28, 33]. To ensure appropriateness and acceptability to CHCs included in our study, the wording of each item was reviewed, and cancer-specific wording was changed to ensure inclusivity. The initial scale consisted of 35 items with respondents indicating their level of need for help in the past month on a four-point Likert scale ranging from no need (1), low need (2), moderate need (3) and high need (4). The USCN survey takes approximately 10 minutes to complete. An exploratory principal component analysis with varimax rotation was conducted (Supplementary tables, Part 1) to determine whether the USCN items could be classified into domains as suggested by others [17, 28, 31]. The scree plot and the eigenvalue > 1 criteria were used to identify the number of factors to retain. Items were eligible to be assigned to a factor if factor loadings were > 0.30. This analysis yielded six factors (termed “domains”) accounting for 70% of the total variance. Cronbach’s alpha coefficient revealed very good internal consistency for the domain subscales derived from identified factors.

Items were rearranged to provide a clear factor solution that was conceptually and clinically relevant [29]. Although items 20, 16, 19 and 33 had greater loadings under other factors, they were retained under factor 4 due to their conceptual or clinical similarity with items on this factor and were primarily designed to assess support needs. Additionally, item 18 did not load conceptually well on any domains and was excluded from subsequent analysis. The domains identified for the 34 items were (1) care needs (7 items, 50.72% variance, *α* = 0.87); (2) physical and social needs (7 items, 4.67% variance, *α* = 0.92); (3) informational needs (4 items, 4.10% variance, *α* = 0.88); (4) support needs (9 items, 3.71% variance, *α* = 0.90); (5) financial needs (3 items, 3.45% variance, *α* = 0.87); and (6) child-related emotional needs (4 items, 3.08% variance, *α* = 0.87) (Supplementary tables, Part 1).

#### Parent and child characteristics

The child’s condition, parent demographic characteristics and treatment-related information were collected. Parent demographic characteristics included age, gender, marital status (partnered vs. other), employment (working vs. not working), and education (up to secondary vs. post-secondary education). Questions also assessed access to a support person such as a partner, friend, or sibling (yes vs. no). Several support organizations for each condition are available and provide free services (e.g., peer support groups, helpline). Respondents indicated that the number of support services (among those listed for each CHC) used was tallied and standardized as the mean score. Child characteristics included sex, current age, disease type, and age at diagnosis and treatment or management (e.g., surgery). Number of hospital visits for professional consultations over the past 12 months was collected with responses recoded into never/once a year, two, and three or more times a year.

### Procedure

Parents were invited to participate through multiple avenues, including advertising on social media platforms and through charity support group organizations. For the latter, organizations specific to the condition (e.g., Redkite, Heart Kids, Diabetes Australia, and Asthma Australia) were contacted, and the study was advertised either through their websites or via email. All invitations provided parents with background information about the study and the link to the online survey. Upon giving consent, parents completed the survey in Qualtrics. Ethics approval was obtained from the institutional review board of Deakin University, Australia (HEAG-H_53_2020).

### Data analysis

Descriptive statistics were used to examine parents demographic characteristics and USCN. The level of need (low, moderate, or high need) endorsed across all items was assessed to identify the proportions reporting no need on all items, low but no moderate/high needs across items, at least one moderate but no high need, or at least one high level need. Need items were binary coded (no/low and moderate/high), and Chi-square analyses assessed differences in the likelihood of indicating a moderate/high need across health conditions. Percentages of moderate/high needs were used to rank the top 5 USCN items. Standardized USCN domain scores were calculated by summing scores for items within the domain, subtracting the number of domain items (indicated as *m*), then multiplying the resulting value by 100/[*m* × (*k* − 1)], where *k* is the maximum response value for each item (i.e., 4; high need) [37]. Standardized domain scores have possible values ranging between 0 and 100, with higher scores indicating greater USCN in that domain. Multiple linear regression analyses examined relationships between each standardized USCN domain score and parents demographic characteristics, child’s age and illness characteristics after controlling for the child’s CHC. Regression analyses were conducted in Stata v17 [38], and all other analyses were conducted in SPSS v28 [39]. Due to low numbers, respondents representing children with asthma were excluded from statistical analyses comparing responses across CHCs. Data from this group are reported in Supplementary tables, Part 2. To assess the impact of including the asthma group in regression analyses, sensitivity analyses involving repeating the multiple regression analyses without the asthma group were conducted.

## Results

### Sample characteristics

Of those accessing the survey site, only 65% completed the survey, and incomplete responses were removed. A total of 201 parents completed the survey. Seven did not meet the inclusion criteria regarding child diagnosis (presence of genetic/developmental conditions) and were removed. Surveys were completed on average approximately 3.3 years post diagnosis of children’s condition, although this varied by condition from around 6 months post-diagnosis for T1D, 1 year for cancer and 5 years for CHD. Of the 194 eligible parents, most were mothers (93%), had a partner (86%) and were aged between 25 and 56 years [mean age = 39, standard deviation (SD) = 6]. With respect to the children with CHCs, 52% were males, aged between 1 and 16 years (mean age = 7, SD = 4). Fifty percent of children had CHD, 26% had T1D, 20% had cancer and 4% had asthma (Table 1 and Supplementary tables, Part 2). Most children with T1D (72%) attended health care professionals three or more times a year, as did 59% of children with cancer and 34% of children with CHD.

**Table 1:** Demographic and clinical characteristics of parents and children with chronic health condition

Variables	Total (*N* = 194)	CHD (*n* = 97)	T1D (*n* = 50)	Cancer (*n* = 39)
Parents				
Age (y)	39.4 (6.0)	38.5 (5.7)	41.3 (5.2)	38.7 (6.8)
Gender^a^				
Male (father)	5.8 (11)	7.2 (7)	6.0 (3)	2.6 (1)
Female (mother)	92.8 (180)	90.7 (88)	92.0 (46)	97.4 (38)
Education^a^				
Up to secondary	37.7 (72)	40.2 (39)	32.0 (16)	35.9 (14)
University degree or more	61.3 (119)	57.7 (56)	66.0 (33)	64.1 (25)
Marital status^a^				
Married or partnered	86.1 (167)	86.6 (84)	80.0 (40)	89.7 (35)
Single (divorced or widowed)	12.4 (24)	11.3 (11)	18.0 (9)	10.3 (4)
Employment^a^				
Working	71.1 (138)	77.3 (75)	66.0 (33)	64.1 (25)
Not working	26.8 (52)	20.6 (20)	32.0 (16)	33.3 (13)
Support^b^				
Yes	84.5 (164)	85.6 (83)	80.0 (40)	84.6 (33)
No	15.5 (30)	14.4 (14)	20.0 (10)	15.4 (6)
Support services used (range 0–100, higher scores greater use)	34.7 (22.0)	28.0 (18.5)	56.4 (20.0)	25.6 (14.8)
Children				
Current age (y)	6.9 (3.7)	6.4 (3.8)	8.1 (3.1)	6.6 (3.9)
Age at diagnosis (y)	3.6 (3.6)	1.2 (1.0)	7.6 (3.2)	4.8 (3.5)
Sex				
Male	52.1 (101)	56.7 (55)	48.0 (24)	43.6 (17)
Female	47.9 (93)	43.3 (42)	52.0 (26)	56.4 (22)
Hospital or health professional attendance				
Never or once/y	34.0 (66)	41.2 (40)	16.0 (8)	41.0 (16)
Twice/y	17.0 (33)	24.7 (24)	12.0 (6)	0.0 (0)
Three or more times/y	49.0 (95)	34.0 (33)	72.0 (36)	59.0 (23)
Treatment and management^c^				
Surgery		92.6 (88)		69.2 (27)
Radiotherapy				17.9 (7)
Chemotherapy				97.4 (38)
Stem cell transplant				7.6 (3)
Taking Insulin via injections or pump			100.0 (50)	
Using CGM			86.0 (43)	
Control medications		48.4 (46)		
Device therapy (e.g., pacemaker)		10.5 (10)		
Cardiac transplant		5.3 (5)		

Data displayed for total sample (*N* = 194) and for CHD, T1D and cancer parents separately. Due to small numbers, data for parents of children with asthma (*n* = 8) are not shown but are provided in Supplementary tables, Part 2. Age, age at diagnosis, support groups used are represented in mean (standard deviation) and all other values are resented in % (*n*). *CHD* congenital heart disease, *T1D* type 1 diabetes mellitus, *CGM* continuous glucose monitor. ^a^Missing cases present; ^b^had at least one person to talk to; ^c^multiple treatment modalities have been used by children

### Prevalence of unmet supportive care needs

Most parents reported at least one unmet need, with 27% reporting at least one moderate and 61% reporting at least one high USCN (Table 2). Parents of children with cancer were most likely to have at least one high USCN (92%), followed by parents of children with T1D (62%). Half of the parents of children with CHD reported at least one high USCN (50%). Mean scores from the standardized domain scales indicate child-related emotional needs the highest and informational needs the lowest USCN across CHCs (Table 2, and Supplementary tables, Part 2).

**Table 2:** Proportion (number) of parents indicating different needs and the mean scores for the standardized domain scales for total sample and for individual health conditions

Variables	Total (*N* = 194)	CHD (*n* = 97)	T1D (*n* = 50)	Cancer (*n* = 39)
Parents with different needs, % (*n*)				
No need^a^	4.1 (8)	8.2 (8)	0.0 (0)	0.0 (0)
Low need^b^	7.7 (15)	11.3 (11)	6.0 (3)	2.6 (1)
Moderate need^c^	27.3 (53)	30.9 (30)	32.0 (16)	5.1 (2)
High need^d^	60.8 (118)	49.5 (48)	62.0 (31)	92.3 (36)
USCN domain^e^, mean (SD)				
Care needs	39.1 (27.0)	30.5 (25.6)	42.6 (28.4)	56.5 (20.2)
Physical and social needs	37.7 (30.5)	27.9 (27.0)	43.1 (31.5)	57.2 (29.3)
Informational needs	32.8 (30.4)	25.4 (27.2)	38.6 (33.0)	46.7 (30.0)
Support needs	41.6 (27.2)	35.8 (24.7)	45.7 (29.8)	55.3 (25.8)
Financial needs	36.0 (33.2)	25.4 (30.0)	50.6 (34.1)	45.8 (30.9)
Child-related emotional needs	46.1 (31.3)	37.8 (28.6)	46.5 (29.8)	71.5 (26.5)

Due to small numbers, data for parents of children with asthma are shown separately in Supplementary tables, Part 2. *CHD* congenital heart disease, *T1D* type 1 diabetes mellitus, *USCN* unmet supportive care needs, *SD* standard deviation. ^a^Selected “no need” on all the 34 items; ^b^selected only low-level needs on items; ^c^at least one moderate need item and no high need items; ^d^at least one “high” need items (may have no, low, moderate needs as well); ^e^range 0–100, higher the scores higher needs

### Commonly reported unmet supportive care needs

Table 3 shows the proportion of parents endorsing “moderate/high” need for each individual need item and displays the top 5 (i.e., most commonly endorsed) USCN for each CHC (except asthma, due to low numbers) and across the overall sample. For most items, the proportions endorsing moderate/high need differed by CHC, with parents of children with cancer significantly more likely to report moderate/high need across the items. The financial items were the exception to this pattern, with more parents of children with T1D and CHD than cancer reporting moderate/high needs. The five most commonly reported USCN in the overall sample were drawn from four need domains: child-related emotional (items ranked 1 and 4), support (items ranked 2 and 3), financial (rank 4), and care (rank 5). Despite the group differences in proportions endorsing moderate/high need on the individual items, the five most commonly endorsed need items were similar across CHCs. Three USCN items were in the top 5 for all CHCs: items “worry about your child’s future” (rank 1), “reducing any stress, you may be experiencing” (rank 2 or 3) and “managing your tiredness or lack of energy” (rank 2, 4 or 5) (Supplementary tables, Part 3).

**Table 3:** Proportion of parents indicating a moderate/high need on each USCN item assessed and five most commonly reported need items for total sample and for individual health conditions

						Five most commonly reported items			
Variables	Total (*N* = 194)	CHD (*n* = 97)	T1D (*n* = 50)	Cancer (*n* = 39)	*χ*^2^ value^a^ (*df* = 2)	Total	CHD	T1D	Cancer
Care needs									
Access to complementary therapy services	43.3 (84)	34.0 (33)	48.0 (24)	64.1 (25)	10.6^†^	5			
Knowing what local health care services might be helpful	28.4 (55)	26.8 (26)	26.0 (13)	38.5 (15)	2.1				
Accessible hospital/health services parking	41.2 (80)	28.9 (28)	40.0 (20)	71.8 (28)	21.2^†^				4
Knowing that health professionals are talking to each other regarding your child’s care	41.2 (80)	30.9 (30)	46.0 (23)	64.1 (25)	13.0^†^				
Managing your child’s health with the health care team	37.1 (72)	28.9 (28)	42.0 (21)	56.4 (22)	9.3^†^				
Knowing what side effects, the treatment can cause	40.7 (79)	20.6 (20)	46.0 (23)	82.1 (32)	44.5^†^				2
Feeling unsure that your child is receiving the best medical care	27.8 (54)	22.7 (22)	38.0 (19)	28.2 (11)	3.8				
Physical and social needs									
Keeping up with your child's school/day-care/kindergarten work	34.5 (67)	25.8 (25)	46.0 (23)	46.2 (18)	8.3*				
Assisting your child to keep up with the things they do	35.6 (69)	28.9 (28)	38.0 (19)	53.8 (21)	7.5*				
Assisting your child to connect with friends	30.4 (59)	22.7 (22)	28.0 (14)	59.0 (23)	17.3^†^				
Managing your child’s tiredness or lack of energy	34.5 (67)	24.7 (24)	40.0 (20)	56.4 (22)	12.7^†^				
Socializing your child with children of their own age	31.4 (61)	25.8 (25)	26.0 (13)	59.0 (23)	15.3^†^				
Supporting your child returning to their usual activities	38.1 (74)	23.7 (23)	50.0 (25)	61.5 (24)	20.4^†^			5	
Managing any side effects your child may be experiencing	42.3 (82)	22.7 (22)	56.0 (28)	71.8 (28)	33.1^†^			3	4
Informational needs									
Having explanations given in a way that you can understand	29.9 (58)	25.8 (25)	30.0 (15)	43.6 (17)	4.1				
Being informed when and why changes were being made in your child’s treatment plans	29.9 (58)	20.6 (20)	34.0 (17)	51.3 (20)	12.6^†^				
Having information about how to care for your child at home	24.7 (48)	17.5 (17)	34.0 (17)	33.3 (13)	6.4*				
Talking honestly with doctors about your child’s future	34.5 (67)	26.8 (26)	38.0 (19)	53.8 (21)	9.0*				
Support needs									
Knowing how to handle the feelings of your other children	42.3 (82)	39.2 (38)	42.0 (21)	59.0 (23)	4.5		4		
Knowing what information to give to your other children (appropriate to his/her age)	30.4 (59)	27.8 (27)	34.0 (17)	38.5 (15)	1.6				
Managing your tiredness or lack of energy	53.6 (104)	48.5 (47)	54.0 (27)	69.2 (27)	4.8	3	2	4	5
Connecting with other parents who have a child with similar conditions	38.7 (75)	29.9 (29)	54.0 (27)	46.2 (18)	8.8*			4	
Information about support services your child could use	41.8 (81)	29.9 (29)	50.0 (25)	66.7 (26)	16.7^†^			5	
Reducing any stress, you may be experiencing	55.2 (107)	47.4 (46)	58.0 (29)	76.9 (30)	9.9^†^	2	3	2	3
Information about support services you could use	38.1 (74)	26.8 (26)	46.0 (23)	61.5 (24)	15.3^†^				
Concerns about how other people will respond to your child once they know they have their illness	31.4 (61)	27.8 (27)	36.0 (18)	38.5 (15)	1.8				
Feeling that the health care professionals were sincere in caring about your child	32.0 (62)	23.7 (23)	32.0 (16)	53.8 (21)	11.5^†^				
Financial needs									
Covering costs of medication for your child	25.8 (50)	12.4 (12)	48.0 (24)	30.8 (12)	22.5^†^				
Paying for your child’s medical care	27.8 (54)	19.6 (19)	42.0 (21)	33.3 (13)	8.7*				
Finding financial support and/or benefits you may be entitled to	44.8 (87)	36.1 (35)	56.0 (28)	56.4 (22)	7.5*	4	5	3	
Child-related emotional needs									
Concerns about your child’s ability to have children in the future	35.6 (69)	23.7 (23)	40.0 (20)	66.7 (26)	22.2^†^				
Fears that your child's illness would progress	44.8 (87)	35.1 (34)	40.0 (20)	82.1 (32)	25.7^†^	4			2
Worry about your child's future	61.3 (119)	51.5 (50)	66.0 (33)	87.2 (34)	15.4^†^	1	1	1	1
Knowing the likely outcomes of your child’s illness	40.7 (79)	33.0 (32)	40.0 (20)	61.5 (24)	9.4^†^				

Data are presented as % (*n*). Data for asthma group not shown separately in table due to small sample size; for interest data can be seen in Supplementary tables, Part 2. *USCN* unmet supportive care needs, *CHD* congenital heart disease, *T1D* type 1 diabetes mellitus. ^a^Chi-square test analyses exclude asthma group. ^*^P < 0.05, ^†^*P* < 0.01

### Factors associated with unmet supportive care needs

After controlling for the child’s diagnosis, only two factors were related to multiple USCN domains: number of times the child attended a hospital/health professional and the absence of a support person (Table 4). A greater number of medical appointments was significantly associated with higher scores for all domains. Except for child-related emotional needs, the absence of a support person was related to higher scores on all domains (Table 4). Children’s age, age at diagnosis, gender, parents age, and employment status were not related to any USCN domains. Other variables were related to one or two USCN domains. The use of support services and parent marital status were positively associated with care needs [*B* = 0.1, standard error (SE) = 0.9, *P* < 0.05] and financial needs (*B* = 14.0, SE = 6.8, *P* < 0.05). Parent education was negatively related to care needs (*B* = − 10.6, SE = 3.8, *P* < 0.01). Sensitivity analyses found no difference in regression results when the asthma group was excluded from the analyses.

**Table 4:** Beta coefficients and standard errors for associations between child and parent factors and scores on each USCN domain (*N* = 194)

Variables	Care needs	Physical and social needs	Informational needs	Support needs	Financial needs	Child-related emotional needs
Child age at diagnosis	− 0.1 (0.9)	− 1.0 (1.0)	0.1 (1.1)	− 0.2 (0.9)	− 1.1 (1.1)	1.2 (1.1)
Child age	− 0.8 (0.6)	1.5 (0.7)	− 0.1 (0.8)	− 0.3 (0.7)	0.2 (0.8)	− 0.4 (0.8)
Child gender-female (1-male, 2-female)	− 4.6 (3.5)	− 5.1 (3.9)	− 3.7 (4.1)	− 1.4 (3.6)	2.9 (4.3)	3.3 (4.1)
Attending hospital						
Twice/y	**15.5 (5.3)**^**†**^	**16.8 (6.1)**^**†**^	**17.0 (6.4)**^**†**^	**18.5 (5.5)**^**†**^	5.4 (6.7)	**(6.3)**^**†**^
Three or more times/y	9.0 (4.1)*	12.0 (4.7)*	12.1 (4.9)*	**11.2 (4.3)**^**†**^	**14.9 (5.2)**^**†**^	9.7 (4.9)*
Parent age	− 0.2 (0.3)	− 0.5 (0.4)	− 0.7 (0.4)	− 0.6 (0.3)	− 0.7 (0.4)	− 0.4 (0.4)
Parent education-university degree (1-upto secondary, 2-university degree or more)	− **10.6 (3.8)**^**†**^	− 1.4 (4.3)	− 4.1 (4.5)	− 4.1 (3.9)	− 0.3 (4.7)	− 7.1 (4.4)
Parent marital status-single (1-partnered, 2-single)	4.2 (5.4)	4.1 (6.1)	− 0.4 (6.5)	2.9 (5.6)	14.0 (6.8)*	1.8 (6.4)
Parent employment-not working (1-working, 2-not working)	1.3 (4.2)	7.7 (4.8)	9.5 (5.0)	3.9 (4.3)	2.9 (5.3)	− 1.2 (5.0)
No support person (1-yes, 2-no)	12.6 (4.9)*	**20.3 (5.6)**^**†**^	13.20 (5.9)*	**15.3 (5.1)**^**†**^	**18.2 (6.2)**^**†**^	5.6 (5.8)
Support service use	0.1 (0.9)*	0.1 (0.1)	0.0 (0.1)	0.0 (0.1)	0.0 (0.1)	0.1 (0.1)
*F* (14,174)	**5.2**^**†**^	**5.2**^**†**^	**3.3**^**†**^	**4.3**^**†**^	**4.2**^**†**^	**4.7**^**†**^
Adjusted *R*^2^	0.24	0.23	0.14	0.19	0.19	0.21

Data are presented as beta coefficients (standard errors). Findings from multiple linear regression with child’s chronic health condition (congenital heart disease, type 1 diabetes, cancer, and asthma) included as covariate. Significant associations are in bold. *USCN* unmet supportive care needs. ^*^P < 0.05, ^†^*P* < 0.01

## Discussion

Using a universal need assessment tool developed for use in families of children affected by some of the most common CHCs, this is one of the first studies to characterize similarities and differences in the SCN of families of children living with a number of different CHCs. With over 60% of parents reporting at least one high USCN, the results suggest that many parents of children with common CHCs experience a gap in the support they need and the support they receive. The proportion of parents identifying a moderate/high need for each item differed between CHCs, with the cancer group having the highest needs, reflecting the different treatment protocols and prognoses of the CHCs included in this study. However, we also found that the types of needs most commonly experienced were similar across CHCs, with “worry about their child’s future”, additional support to help “reduce their own stress”, and “manage tiredness” as key issues for parents. These findings suggest that some areas of support could be generalized across CHCs. A greater frequency of visits to hospital/medical appointments was associated with a higher need across domains, suggesting the role that medical demands, treatment or lack of health information and self-care skills have in generating unmet needs for parents [14, 40, 41]. Additionally, we found that the absence of a support person was associated with a higher USCN in five of the six domains. Previous studies have also shown that the lack of a support person can contribute to greater care burdens for parents [42, 43]. These findings provide suggestions for factors that might help identify parents who require additional assistance regardless of their child’s health condition.

The high prevalence of unmet needs across conditions indicates that many parents require more support than they are currently accessing. While pediatric health services in Australia provide psychology and social work services, access may be hindered due to variations in the type of services available, eligibility criteria, a lack of coordinated care, and limited information about services [44]. Community-based support organizations for each condition provide access to additional support services. However, the use of these services was relatively low in our study, with the highest usage found among parents of children with T1D. As others have noted, work to identify, and implement effective strategies to better link parents to relevant services in the community and at health services is needed [10, 15, 26].

Apart from the literature relating to pediatric cancer, few studies have assessed the unmet SCN of families of children with CHD, T1D or other conditions to compare our findings [33]. We found that parents need for additional support was high across all CHCs. Previous pediatric cancer and CHD studies reported high levels of emotional needs in parents, with most reporting needs regarding managing anxiety, stress, or grief [19, 25, 30, 32, 45, 46]. Our study also found that needs relating to managing concerns about the future or illness progression were common across CHCs. While this domain has been identified by others examining the needs of families of children with cancer [32], our recent systematic review suggests that child-related emotional concerns are often combined with general parental emotional concerns, including feelings of anxiety, depression, and grief [33]. The integration of needs relating to the carer’s own emotional state and concerns about the patient’s disease progression into a single emotional need domain is also seen in the adult carer USCN literature [24, 29, 47]. Although the need domains identified in our study are broadly consistent with the SCN framework, the high frequency of financial needs in the T1D group suggests that work is required to ensure that all relevant unmet need domains are captured by assessment tools. In addition, although needs relating to school, communication and lifestyle management have been identified [48–50], their relevance across childhood CHCs is less well understood and requires further investigation.

Regardless of the child’s condition, two commonly reported USCN in our study were reducing parents stress and managing tiredness. Stress in parents caring for a child with a CHC is common and is unrelated to illness duration or severity [12]. Many factors have been associated with parenting stress, including treatment adherence/management responsibilities, frequent hospitalizations, communication with health care providers, disruption of a normal family life, and lack of resources or social support [12, 51]. Need items reflecting these factors were reported as unmet in our study. Our findings suggest that many parents of children with CHCs require more assistance in managing the stress they experience.

The need for financial support/assistance was endorsed by many families and was a top 5 unmet needs for families of children with T1D and CHD. Financial burden has been identified previously [46, 52–56]. Studies have shown that many mothers of children with one of our health conditions restrict their hours of paid work to care for their child [54], which may add to the financial impact of these conditions. In addition, ongoing costs associated with T1D management may contribute to a significant financial burden for families [54]. Similarly, in CHD, lack of insurance coverage and disease complexity/treatment costs over time can contribute to increased financial burden [55, 56]. While parents of children with cancer also experience financial burden [57] in our study, the proportions reporting a financial need were less than T1D and CHD parents. In the Australian context, this finding may reflect that pediatric cancer care is primarily delivered in the public hospital system, which reduces out-of-pocket costs for families. While there is government assistance for T1D care, including devices, this funding may not cover all costs. The lifelong need for T1D care may place additional stress on parents. With few studies examining the financial needs of parents of children with CHCs, our study suggests that work is needed to identify the type and drivers of financial needs parents have.

Although parents of children with CHD were less likely to experience moderate/high needs than parents of children with cancer or T1D, our study found that 80% experienced at least one moderate/high need for additional support. Previous research in parents of children with CHD found similar results, with 59% having at least one need and 40% of parents reporting a need for psychosocial care [45, 46]. Despite differences in the timing of our survey and treatment stage (from diagnosis to 5 years post-treatment), the level of USCN found in previous studies is similar to the levels reported here. Additional work is required to identify and classify the unique needs of parents across the trajectory of CHD care.

This is one of the few studies to use the same assessment tool to examine unmet needs across different CHCs post-acute treatment, allowing comparison of type and extent of needs across conditions. The initial psychometric evaluation of this newly established assessment tool showed good reliability but requires further validation. Recruitment via online platforms (including social media platforms associated with condition-specific support services and charity organizations) and selection bias (mostly mothers) may limit the potential generalizability of the findings. It is possible that those who engage with these platforms may have greater needs [58]. Recruitment of parents of children at various stages of care and during the coronavirus disease 2019 (COVID-19) pandemic might have caused this disparity and elevated needs. The sample size of parents of children with different CHCs varied considerably, with low numbers for asthma. While this may reflect a lack of need among these different groups, it is also possible that the timing of the survey influenced participation with parents focusing on managing their child’s health during the COVID-19 pandemic [59, 60]. Finally, our survey was based on measures developed for cancer populations. While the similarities in the ranking of needs across the CHCs suggest the utility of this approach, further work is needed to determine a suitable tool to assess USCN across multiple CHCs. The findings from this study suggest that this work is warranted.

In conclusion, our results show that a significant number of parents caring for a child with a CHC have a moderate/high level of needs, demonstrating a gap in the support services they require and receive. As there is a lack of intervention studies addressing the USCN of pediatric CHCs, future research should develop and test interventions. The similarity of the top ranked needs across CHCs suggests that support programs, particularly those that aim to help parents address their fatigue and stress, could be shared across health conditions. To assist this work, research is also needed to develop a standardized measure for assessing the USCN of parents across multiple CHCs.

## Supplementary Information

Below is the link to the electronic supplementary material.

Supplementary file 1 (PDF 199 kb)

## Data Availability

Due to ethical and privacy reasons data is not publicly available. Datasets generated during the current study will be accessible from corresponding author upon reasonable request.
