# Supplementary material for: Unmet supportive care needs in families of children with chronic health conditions: an Australian cross-sectional study
Source: World J Pediatr. 2023 May 29;19(12):1181–91. doi: 10.1007/s12519-023-00730-w (PMC10225288; doi:10.1007/s12519-023-00730-w)
Supplement: Supplementary file 2 — (PDF 199 kb) [file 12519_2023_730_MOESM2_ESM.pdf]

Part 1: Data generated from the unmet supportive care needs survey

Supplementary Table 1.1 Factor loading of principal component analysis using varimax rotation of unmet supportive care needs survey

Rotated component matrix[a]	Component					
	1	2	3	4	5	6
**Factor 1: care needs**						
Item 10: access to complementary therapy services	0.683					
Item 4: knowing what local health care services might be helpful	0.575		0.332	0.308		
Item 7: accessible hospital/health services parking	0.543	0.341				
Item 6: knowing that health professionals are talking to each other regarding your child’s care	0.538		0.534			
Item 5: managing your child’s health with the health care team	0.528	0.402	0.442			
Item 8: knowing what side effects the treatment can cause	0.524	0.441	0.371			0.312
Item 3: feeling unsure that your child is receiving the best medical care	0.480		0.385			
Item 18: worry about partners, family, and friends	0.404	0.368				
**Factor 2: physical and social needs**						
Item 24: keeping up with your child's school/day-care/ kindergarten work		0.790				
Item 23: assisting your child to keep up with the things they do		0.741				
Item 25: assisting your child to connect with friends		0.712		0.455		
Item 22: managing your child’s tiredness or lack of energy		0.671				0.319
Item 26: socializing your child with children of their own age	0.317	0.610		0.465		
Item 17: supporting your child returning to their usual activities	0.318	0.510		0.325		0.302
Item 9: managing any side effects your child may be experiencing	0.361	0.507				0.355
**Factor 3: informational needs**						
Item 34: having explanations given in a way that you can understand			0.787			
Item 35: being informed when and why changes were being made in your child’s treatment plans			0.751			
Item 32: having information about how to care for your child at home		0.363	0.628			
Item 21: talking honestly with doctors about your child’s future	0.300	0.367	0.495	0.341		0.369
**Factor 4: support needs**						
Item 29: knowing how to handle the feelings of your other children				0.654		

Item 30: knowing what information to give to your other children (appropriate to his/her age)			0.307	0.634		
Item 28: managing your tiredness or lack of energy	0.392			0.593		0.360
Item 27: connecting with other parents who have a child with similar conditions				0.567		
Item 20: information about support services your child could use	0.537			**0.499**		
Item 16: reducing any stress, you may be experiencing	0.548			**0.456**	0.336	0.347
Item 19: information about support services you could use	0.547		0.376	**0.446**		
Item 11: concerns about how other people will respond to your child once they know they have their illness	0.328			0.378	0.348	
Item 33: feeling that the health care professionals were sincere in caring about your child			0.675	**0.303**		
**Factor 5: financial needs**						
Item 13: covering costs of medication for your child					0.849	
Item 14: paying for your child’s medical care					0.823	
Item 12: finding financial support and/or benefits you may be entitled to	0.425				0.658	
**Factor 6: child-related emotional needs**						
Item 15: concerns about your child’s ability to have children in the future					0.372	0.674
Item 1: fears that your child's illness would progress	0.421		0.305			0.653
Item 2: worry about your child's future	0.522					0.565
Item 31: knowing the likely outcomes of your child’s illness			0.402	0.437		0.534

Rotation method: varimax with Kaiser normalization. Extraction method: principal component analysis; irrespective of greater loading under other factors for items 20, 16, 19 and 33 they were retained under factor 4 (these loadings are in bold). Item 18 did not load conceptually well on any factors and was excluded. [a]Rotation converged in 14 iterations

Supplementary Table 1.2. Unmet supportive care needs survey

	For every item listed below, *please indicate whether you have needed help or would have liked help with this issue within the last month as a result of your child's health condition*. Select the response that best describes whether you have needed help with this issue in the last month. If you would have liked help in these areas in the past month, please indicate if this need was low, moderate or high	**No need (1)**	**Low need (2)**	**Moderate need (3)**	**High need (4)**
	**Care needs**				
1	Access to complementary therapy services				
2	Knowing what local health care services might be helpful				
3	Accessible hospital/health services parking				
4	Knowing that health professionals are talking to each other regarding your child's care				
5	Managing your child's health with the health care team				
6	Knowing what side effects the treatment can cause				
7	Feeling unsure that your child is receiving the best medical care				
	**Physical and social needs**				
8	Keeping up with your child's school/day-care/ kindergarten work				
9	Assisting your child to keep up with the things they do				
10	Assisting your child to connect with friends				
11	Managing your child's tiredness or lack of energy				
12	Socializing your child with children of their own age				
13	Supporting your child returning to their usual activities				
14	Managing any side effects your child may be experiencing				
	**Informational needs**				
15	Having explanations given in a way that you can understand				
16	Being informed when and why changes were being made in your child's treatment plans				
17	Having information about how to care for your child at home				
18	Talking honestly with doctors about your child's future				
	**Support needs**				
19	Knowing how to handle the feelings of your other children				
20	Knowing what information to give to your other children (appropriate to his/her age)				
21	Managing your tiredness or lack of energy				

22	Connecting with other parents who have a child with similar conditions
23	Information about Support Services your child could use
24	Reducing any stress, you may be experiencing
25	Information about support services you could use
26	Concerns about how other people will respond to your child once they know they have their illness
27	Feeling that the health care professionals were sincere in caring about your child
	**Financial needs**
28	Covering costs of medication for your child
29	Paying for your child’s medical care
30	Finding financial support and/or benefits you may be entitled to
	**Child-related emotional needs**
31	Concerns about your child’s ability to have children in the future
32	Fears that your child's illness would progress
33	Worry about your child's future
34	Knowing the likely outcomes of your child’s illness

Part 2: Data generated from the asthma group

Supplementary Table 2.1. Demographic and clinical characteristics of parents and children with asthma (*n* = 8)

Variables	Values
Parents	
Age	41.1 (6.9)
Gender[a]	
Male (father)	0
Female (mother)	8
Education[a]	
Up to secondary	3
University degree or more	5
Marital status[a]	
Married or partnered	8
Single (divorced or widowed)	0
Employment[a]	
Working	5
Not working	3
Support[b]	
Yes	8
No	0
Support services used (range: 0-100, higher scores greater use)	25.0 (9.2)
Children	
Current age	6.3 (4.1)
Age at diagnosis	1.8 (0.6)
Sex	

Male	5
Female	3
Hospital or health professional attendance	
Never or once a year	2
Twice a year	3
Three or more times a year	3
Treatment and management[c]	
Control medications	8
Device therapy (e.g., inhaler)	5
Asthma plan	8
Asthma diary	3

Age, age at diagnosis, support groups used are represented in mean (standard deviation) and all other values are represented as frequency (*n*).

[a]Missing cases present; [b]had at least one person to talk to; [c]multiple treatment modalities have been used by children

Supplementary Table 2.2. Number of parents indicating no needs at all, only low needs, only moderate needs and any high needs and the mean scores for the standardized domain scales for asthma group

**Variables**	**Values**
Number of parents with different needs, *n*	
No need[a]	0
Low need[b]	0
Moderate need[c]	5
High need[d]	3
USCN domain[e], mean (SD)	
Care needs	35.7 (21.8)
Physical and social needs	26.7 (14.3)
Informational needs	18.7 (24.7)

Support needs	19.9 (18.5)
Financial needs	25.0 (30.7)
Child-related emotional needs	20.8 (23.5)

USCN unmet supportive care needs, *SD* standard deviation. [a]Selected "no need" on all the 34 items; [b]selected only low-level needs on items; [c]at least one moderate need item and no high need items, [d]at least one "high" need items (may have no, low, moderate needs as well); [e]range 0-100, higher the scores higher needs

Supplementary Table 2.3. Number of parents of children with asthma indicating a moderate/high need on each USCN item assessed (*n* = 8)

Variables	*n*
**Care needs**	
Access to complementary therapy services	2
Knowing what local health care services might be helpful	1
Accessible hospital/health services parking	4
Knowing that health professionals are talking to each other regarding your child's care	2
Managing your child's health with the health care team	1
Knowing what side effects, the treatment can cause	4
Feeling unsure that your child is receiving the best medical care	2
**Physical and social needs**	
Keeping up with your child's school/day-care/ kindergarten work	1
Assisting your child to keep up with the things they do	1
Assisting your child to connect with friends	0
Managing your child's tiredness or lack of energy	1
Socializing your child with children of their own age	0
Supporting your child returning to their usual activities	2
Managing any side effects your child may be experiencing	4
**Informational needs**	
Having explanations given in a way that you can understand	1
Being informed when and why changes were being made in your child's treatment plans	1
Having information about how to care for your child at home	1
Talking honestly with doctors about your child's future	1
**Support needs**	
Knowing how to handle the feelings of your other children	0
Knowing what information to give to your other children (appropriate to his/her age)	0

Managing your tiredness or lack of energy	3
Connecting with other parents who have a child with similar conditions	1
Information about support services your child could use	1
Reducing any stress, you may be experiencing	2
Information about support services you could use	1
Concerns about how other people will respond to your child once they know they have their illness	1
Feeling that the health care professionals were sincere in caring about your child	2
**Financial needs**	
Covering costs of medication for your child	2
Paying for your child’s medical care	1
Finding financial support and/or benefits you may be entitled to	2
**Child-related emotional needs**	
Concerns about your child’s ability to have children in the future	0
Fears that your child's illness would progress	1
Worry about your child's future	2
Knowing the likely outcomes of your child’s illness	3

USCN unmet supportive care needs

Part 3: Ranking of needs

Supplementary Table 3.1. Ten most commonly reported moderate/high USCN items (and domain) for parents of children with congenital heart disease, type 1 diabetes and cancer (*n* = 186) (ranked by percentage within each condition)

	**CHD**		**T1D**		**Cancer**	
**Rank**	**Item**	**Domain**	**Item**	**Domain**	**Item**	**Domain**
1	Worry about your child's future	Child related-emotional needs	Worry about your child's future	Child related-emotional needs	Worry about your child's future	Child related-emotional needs
2	Managing your tiredness or lack of energy	Support needs	Reducing any stress, you may be experiencing	Support needs	Knowing what side effects, the treatment can cause<br>Fears that your child's illness would progress	Care needs<br>Child related-emotional needs
3	Reducing any stress, you may be experiencing	Support needs	Managing any side effects your child may be experiencing<br>Finding financial support and/or benefits you may be entitled to	Physical and social needs<br>Financial needs	Reducing any stress, you may be experiencing	Support needs
4	Knowing how to handle the feelings of your other children	Support needs	Connecting with other parents who have a child with similar conditions<br>Managing your tiredness or lack of energy	Support needs	Managing any side effects your child may be experiencing<br>Accessible hospital/health services parking	Physical and social needs<br>Care needs

5	Finding financial support and/or benefits you may be entitled to	Financial needs	Information about support services your child could use<br>Supporting your child returning to their usual activities	Support needs<br>Physical and social needs	Managing your tiredness or lack of energy	Support needs
6	Fears that your child's illness would progress	Child related-emotional needs	Covering costs of medication for your child<br>Access to complementary therapy services	Financial needs<br>Care needs	Information about support services your child could use<br>Concerns about your child's ability to have children in the future	Support needs<br>Child related-emotional needs
7	Access to complementary therapy services	Care needs	Information about support services you could use<br>Knowing what side effects, the treatment can cause<br>Knowing that health professionals are talking to each other regarding your child's care<br>Keeping up with your child's school/day-care/kindergarten work	Support needs<br>Care needs<br>Care needs<br>Physical and social needs	Knowing that health professionals are talking to each other regarding your child's care<br>Access to complementary therapy services	Care needs<br>Care needs
8	Knowing the likely outcomes of your child's illness	Child related-emotional needs	Managing your child's health with the health care team<br>Knowing how to handle the feelings of your other children<br>Paying for your child's medical care	Care needs<br>Support needs<br>Financial needs	Knowing the likely outcomes of your child's illness<br>Information about support services you could use<br>Supporting your child returning to their usual activities	Child related-emotional needs<br>Support needs<br>Physical and social needs

9	Knowing that health professionals are talking to each other regarding your child's care	Care needs	Managing your child's tiredness or lack of energy<br>Accessible hospital/health services parking<br>Concerns about your child's ability to have children in the future<br>Fears that your child's illness would progress<br>Knowing the likely outcomes of your child's illness	Physical and social needs<br>Care needs<br>Child related-emotional needs<br>Child related-emotional needs<br>Child related-emotional needs	Knowing how to handle the feelings of your other children<br>Socializing your child with children of their own age<br>Assisting your child to connect with friends	Support needs<br>Physical and social needs<br>Physical and social needs
10	Connecting with other parents who have a child with similar conditions<br>Information about support services your child could use	Support needs	Talking honestly with doctors about your child's future<br>Feeling unsure that your child is receiving the best medical care<br>Assisting your child to keep up with the things they do	Informational needs<br>Care needs<br>Physical and social needs	Finding financial support and/or benefits you may be entitled to<br>Managing your child's health with the health care team<br>Managing your child's tiredness or lack of energy	Financial needs<br>Care needs<br>Physical and social needs

Ranking has not been provided for asthma due to small sample size and equal rankings. *USCN* unmet supportive care needs, *CHD* congenital heart disease, *T1D* type 1 diabetes mellitus
